# Interferon Alpha on Expression of hTERT mRNA in Peripheral Blood Mononuclear Cells of Patients with Chronic Hepatitis B

**DOI:** 10.1155/2011/920146

**Published:** 2011-05-15

**Authors:** Chuan-wu Zhu, Ming Chen, Xiang-rong Luo, Hai-yan Wang, Li-hua Wang, Jian-hong Wu, Ming Li, Xue-hua Zhang, Wei Zhu, Jian-zhong Ye, Feng Qian

**Affiliations:** ^1^Department of Hepatology, The Fifth People's Hospital of Suzhou, Suzhou 215007, China; ^2^Department of Infectious Diseases, Affiliated Hospital of Medical College of Xuzhou, Xuzhou 221000, China

## Abstract

Cell division is closely related to telomerase activity (hTERT mRNA). Lower expression of lymphocitic hTERT mRNA may easily cause cell aging, which is not beneficial to maintaining a durable lymphocyte division. To date, there is no study to investigate IFN*α* therapy on hTERT mRNA expression in PBMCs of patients with chronic hepatitis B (CHB). We quantitatively detected hTERT mRNA from study subjects and made each hTERT mRNA normalized (NhTERT mRNA). Mean NhTERT mRNA level was lower in either CHB group, but it significantly increased in IFN*α*-treated group compared with CHB control group, and a longer duration of IFN*α* therapy could increase the level. Moreover, the mean NhTERT mRNA in subgroup with HBeAg loss was significantly higher than that in subgroup without. NhTERT mRNA was markedly correlated with CD3^+^ T lymphocyte count and CD4^+^/CD8^+^ ratio. The results showed that IFN*α* therapy could upregulate the expression of hTERT mRNA in PBMCs.

## 1. Introduction

Telomeres are the terminal repeats of the chromosomes in eukaryotic cells and continuously shorten with cell division. Acting as a molecular clock, telomeres control the capacity of cell division and have a close relationship with cell senescence [1]. Telomerase is an RNA-dependent DNA polymerase, maintains the telomere length, and makes the cell achieve unlimited proliferation capacity [2]. Telomerase consists of two subunits, namely, functional RNA component (human telomerase RNA, hTR) and human telomerase reverse transcriptase catalytic subunit (hTERT) [3, 4]. hTR, which is highly expressed in all tissues regardless of telomerase activity, provides telomerase with the template for the synthesis of telomeres, while the expression of hTERT is in accordance with telomerase activity [5, 6]. Therefore, the level of hTERT mRNA could reflect the activity of telomerase. Germ cells and tumor cells often express higher level of telomerase activity, whereas most of normal somatic cells do not [7, 8], indicating that telomerase expression is closely related to the cell differentiation and development. The lymphocytes among somatic cells, however, can produce comparatively higher level of telomerase activity [9, 10], which can keep telomere length of lymphocytes from shortening, and thus play an important role in maintaining a durable cell differentiation. If the lymphocytes express low level of telomerase activity, telomere length will gradually shorten with cell division and finally result in cell aging and immune dysfunction, which has been confirmed in the researches of human immunodeficiency virus (HIV) and chronic hepatitis B virus (HBV) infection [11, 12]. Up to now, little is known about the alteration of telomerase activity in lymphocytes during the antiviral therapy on patients with chronic hepatitis B (CHB). We herein quantitatively detected the expression of hTERT mRNA in peripheral blood mononuclear cells (PBMCs) from CHB patients who received interferon alpha (IFN*α*) treatment and attempted for the first time to study the possible relationship between the immunomodulator and the telomerase activity in PBMCs.

## 2. Materials and Methods

### 2.1. Study Subjects

All investigated CHB patients, who met the criteria of the guidelines of prevention and treatment for chronic hepatitis B issued by Chinese Society of Hepatology [13], were those hospitalized in the Fifth People's Hospital of Suzhou during the year of 2009. The study was composed of three groups, namely, IFN*α*-treated group, CHB control group, and healthy control group. None of the patients in IFN*α*-treated group and CHB control group received any immunomodulator treatment including immune enhancers or immunosuppressive agents, as well as antiviral agents including nucleoside/nucleotide analogues for at least 6 months before IFN*α* therapy initiated. All patients were ruled out the coinfection with hepatitis A, C, D, or E viruses, as well as other liver damages caused by alcohol, drug, autoimmunity, and so forth. Healthy control group consisted of our 10 volunteer colleagues, who had normal liver function and were tested negative for the serum immunological markers of hepatitis B, C, and D virus infection. All subjects gave their voluntary informed consents about the study, and the research protocol was approved by the ethics committee of the Fifth People's Hospital of Suzhou. In IFN*α*-treated group, all the patients were given conventional IFN*α*-2b 5 MU intramuscular injection once a day for 2 weeks, and then were injected every other day for the total duration of 1 year. During the treatment, liver function and blood cell counts were tested monthly, and HBV serological markers and HBV DNA load were tested every 3 months. 

### 2.2. Baseline Clinical Tests

In IFN*α*-treated group and CHB control group, all the baseline HBV serological markers (Abbott Ireland, Diagnostics Division, Sligo, Ireland), HBV DNA loads (Shanghai Shenyou Technology Co. Ltd, Shanghai, China), HBV genotypes (Shanghai ZJ Biological Technology Co. Ltd, Shanghai, China), and the routine biochemical tests, including liver function, kidney function, thyroid function, blood sugar, and peripheral blood cell counts, and so forth were detected. Lymphocyte subsets (BD Biosciences, San Jose, CA, USA) in 28 cases of IFN*α*-treated group and 26 cases of CHB control group were detected at baseline, and the lymphocyte subsets were retested in 26 patients after given IFN*α* treatment for at least 6 months. 

### 2.3. PBMCs Sample Collection

5 mL of heparin-anticoagulated peripheral blood was collected from each subject. Among them, the samples from IFN*α*-treated group were collected at the time of 6, 9, and 12 months of treatment, and those from CHB control group were collected on the second day of the patient's hospitalization. PBMCs were isolated by standard Ficoll density-gradient centrifugation and then washed with 0.01 M phosphate buffer solution (PBS). After centrifugation, about 5 × 10^6^ cells were gathered for each sample and kept frozen at −80°C until the samples were used.

### 2.4. Total Cellular RNA Extraction

Total cellular RNA was, respectively, extracted using BIOZOL (Hangzhou Bioer Technology Co. Ltd, Hangzhou, China) according to the manufacturer's instruction. Briefly, each PBMCs sample was, respectively, precipitated and lysed, and then the cellular RNA was isolated, precipitated, washed, and finally dissolved in 30 *μ*L of RNase-free water. The integrity of RNA was determined, respectively. The samples with obvious 28S and 18S rRNA components were used for the following study.

### 2.5. Detection of hTERT mRNA and *β*-actin mRNA

hTERT mRNA was quantitatively detected by real-time reverse transcription polymerase chain reaction (RT-PCR) (AB Applied Biosystems 7300 real-time PCR System, USA), using the quantification kit (Hangzhou Bioer Technology Co. Ltd, Hangzhou, China) conducted in accordance with the product instruction. Total reactive volume was 40 *μ*L and contained 4 *μ*L of RNA sample (1 *μ*g or so). After 40 cycles of reaction, the quantitative results could be obtained automatically from PCR detection system, and the unit of hTERT mRNA is copies/ml in terms of the kit indication. In order to exclude all the possible factors influencing the comparison of hTERT mRNA levels among different samples, *β*-actin was used as internal control and its mRNA was also quantitatively detected by real-time RT-PCR for each sample, using the quantification kit (Shanghai Genepharma Co. Ltd, Shanghai, China) performed according to the manufacturer's instruction. The results also could be obtained automatically and the unit is copies/*μ*l. Therefore, a normalized hTERT mRNA (NhTERT mRNA) could be obtained for each sample; that is, NhTERT mRNA could be calculated as the copies of target gene hTERT mRNA divided by the copies of its internal control gene *β*-actin mRNA.

### 2.6. Statistical Analysis

A descriptive analysis of the variables collected from each subject was performed. Continuous variables were shown as median values and the dispersion measurement as range values. The chi-squared test and *t*-test were used for analysis of the selected variables. Mean NhTERT mRNA level was presented as mean ± SD. The differences between two groups were compared using independent-samples *t*-test and multiple groups using one-way analysis of variance. Spearman rank correlation analysis was used for bivariate correlation analysis. A probability value was determined using two-sided statistical test. *P* < .05 was considered to be statistically significant. All statistical analyses were performed using SPSS version 13.0 for windows (SPSS, Chicago, IL, USA).

## 3. Results

### 3.1. Baseline Characteristics of the Study Groups

The clinical characteristics of the three groups were listed in Table 1. Gender and age were listed for all groups. HBeAg status, aniline aminotransferase (ALT) levels, HBV DNA levels, and HBV genotypes were listed for all CHB patients. The counts of CD3 and the ratio of CD4^+^/CD8^+^ were listed for 28 patients in IFN*α*-treated group and 26 patients in CHB control group. There was/were no significant difference(s) for these baseline characteristics among the study groups. 

### 3.2. IFN*α* Therapy and Responses

In IFN*α*-treated group, the results of change in biochemical, immunological, and virological indices were evaluated after the beginning of antiviral therapy for at least 6 months. In this study, there were 11 patients evaluated at the time of treatment for 6 months, 14 patients for 9 months, and 12 patients for 12 months. In the beginning of IFN*α* therapy, the varying degrees of flu-like symptoms occurred, the count of neutrophils and platelets decreased in some patients, and no severe adverse events which led to IFN*α* dose reduction or antiviral interruption happened. The biochemical, virological, and immunological responses to IFN*α* were as follows: ALT normalization (<40 IU/mL) in 23 cases, ALT elevation (≥40 IU/mL) in 14 cases; HBV DNA load <3 log/mL in 18 cases, HBV DNA load ≥3 log/mL in 19 cases; and among 31 cases of HBeAg positive patients, 14 cases with HBeAg loss, and 17 still with positive serum HBeAg.

### 3.3. Comparison of Mean NhTERT mRNA Levels

**Figure 1:**
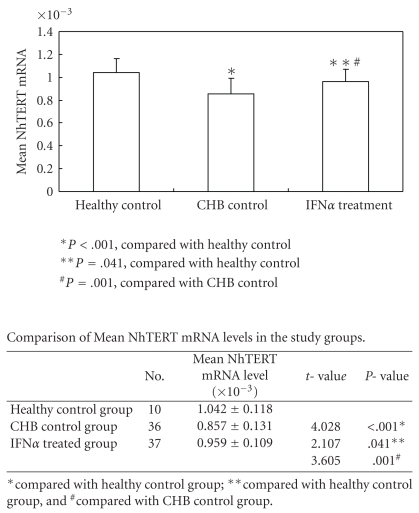
Mean NhTERT mRNA levels in the study groups.

**Figure 2:**
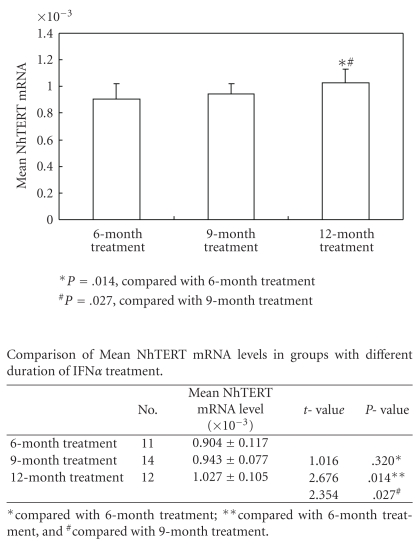
Mean NhTERT mRNA levels in groups with different duration of IFN*α* treatment.

Each level of NhTERT mRNA in PBMCs of the three groups could be seen in Table 2. Among the three groups, the mean NhTERT mRNA was 1.042 × 10^−3^ in healthy control group, 0.959 × 10^−3^ in IFN*α*-treated group, and 0.857 × 10^−3^ in CHB control group, respectively. The compared results were shown in Figure 1. There were no remarkable differences between different gender groups, genotype groups, as well as HBeAg positive and negative groups (*P* = .753, *P* = .664, and *P* = .587, resp.). Regarding the duration of IFN*α* treatment on the influence of mean NhTERT mRNA levels, Figure 2 showed that the patients with longer duration of IFN*α* treatment had higher levels of NhTERT mRNA in PBMCs.

### 3.4. Relationship of NhTERT mRNA with Clinical Characteristics

Spearman rank correlation analysis showed that the NhTERT mRNA expression was not associated with age in all subjects (*r* = −.059, *P* = .594, *n* = 83), and neither was with ALT in all CHB patients (*r* = −.116, *P* = .329, *n* = 73). There was significant relationship between the expression of NhTERT mRNA and the count of CD3^+^ T lymphocytes (*r* = .304, *P* = .026, *n* = 54) as well as the ratio of CD4^+^/CD8^+^ (*r* = .285, *P* = .041, *n* = 54) when the 54 patients with lymphocyte subset test were taken into account as a total and analyzed. And among them, 26 patients in IFN*α*-treated group had their lymphocyte subset retested, and further analysis showed that the level of NhTERT mRNA was much more significantly associated with the count of CD3^+^ T lymphocytes (*r* = .572, *P* = .003, *n* = 26) and so was with the ratio of CD4^+^/CD8^+^ (*r* = .527, *P* = .008, *n* = 26). In addition, there were 25 patients with detectable serum HBV DNA in IFN*α* treated group (≥2.7 log copies/mL based on the lowest limit of the RT-PCR kit), and analysis showed that NhTERT mRNA level was not significantly correlated with HBV DNA load whether in CHB control group (*r* = .230, *P* = .176, *n* = 36) or in IFN*α* treated group (*r* = −.209, *P* = .316, *n* = 25). 

### 3.5. Relationship between NhTERT mRNA and Antiviral Responses

The patients in IFN*α*-treated group were further divided into three subgroups in terms of their biochemical, virological, and immunological responses to IFN*α* therapy. We found that the mean NhTERT mRNA level was significantly higher in HBeAg loss subgroup compared with HBeAg positive subgroup (*P* = .015), and especially, the level in HBeAg loss subgroup was comparable to that in healthy control group (*P* = .479). The mean NhTERT mRNA level was higher in HBV DNA load ≤3 log subgroup compared with HBV DNA load >3 log subgroup but did not reach statistical significance (*P* = .059). There was no significant difference between ALT normal and abnormal subgroups (*P* = .249, Table 3). 

## 4. Discussion

Lymphocytes are indispensable components for the host immune response, and the enough number of the cells is the basic precondition to maintain the normal immune function. During the typical immune reaction, the proliferation of lymphocytes increases 10^5^-10^6^ fold compared with the resting state [14]. The cellular replicative life span in normal somatic cells is finite. However, lymphocytes are the rare somatic cells to obtain the relatively enduring ability to divide, which may be, to some extent, associated with lymphocytic telomere length, because the cell replicative capacity depends largely on their telomere length. The length of human telomeric repeats is 5–15 kb [15, 16] and shortens gradually with cell division. When telomeres shorten to a certain limit, cells cannot replicate anymore and chromosomal instability and cell senescence will ensue [17]. The shortening telomere length of lymphocytes is a sign of immune senescence [18]. Telomerase can synthesize the repeat sequence TTAGGG at the telomeric ends and which can resist the shortening of telomere length; so the lymphocytes with higher telomerase activity should have more durable ability for cell division and proliferation. The researches have confirmed that the antigen-specific T lymphocytes can maintain telomere length so long as telomerase activity is high [19, 20]. In the study of HIV-infected patients, the expression of hTERT mRNA in CD4^+^ T lymphocytes was found significantly decreased [21, 22], which could lead to a reduced telomerase activity and shortened cell life expectancy and thus result in a limited proliferation of CD4^+^ T cell clones. That the lymphocytes possess a lower hTERT mRNA level may also be responsible for the decreasing number of CD4^+^ T lymphocytes besides the HIV direct injury to the cells. Therefore, based on the performed studies, we can find that a certain relationship exists between the telomerase activity and the immune cells. 

In our study, we quantitatively detected hTERT mRNA in PBMCs from CHB patients and healthy controls. However, it was not suitable to directly compare the hTERT mRNA level among the subjects mainly because of the two reasons: (1) the count of PBMCs was not absolutely equal for each sample; (2) the RNA concentration was difficult to keep equal in the management of cellular RNA extraction for each sample. Therefore, in order to reasonably analyze the data, *β*-actin, a house-keeping gene which expresses invariably in all tissues and cells, was used as internal control and its mRNA was also quantitatively detected. So a relative comparison value, NhTERT mRNA, was obtained, and which reflects a relative level of hTERT expression as one copy of *β*-actin mRNA is expressed in PBMCs. The relative value should be stable for a certain individual and could be analyzed reasonably among the subjects. Of course, it would be much better to directly analyze hTERT mRNA if the count of isolated lymphocytes and the concentration of extracted RNA could be obtained equally for each subject. From our data, we found that the expression of NhTERT mRNA in PBMCs of CHB patients presented significantly positive correlation with both the count of CD3^+^ T lymphocytes and the ratio of CD4^+^/CD8^+^, and moreover, the correlation extent seemed to be more remarkable for these indices in IFN*α*-treated group. NhTERT mRNA level did not show the significant correlation with the age of subjects, patient's ALT level, and the load of HBV DNA, respectively. The mean level of NhTERT mRNA was also not significantly different among the different HBV genotype groups. The results showed that the lymphocytic telomerase activity was closely associated with the number of lymphocytes and the immune function in CHB patients.

In vitro studies, it had been found that the telomerase activity significantly increased when the peripheral blood T lymphocytes were stimulated with mitogens, such as phytohemagglutinin (PHA) or phorbol ester (PMA), anti-CD3 antibody, interleukin-2 (IL-2), and other cytokines, but significantly decreased telomerase activity could be caused by use of hydrocortisone stimulating PBMCs [6, 23–25], indicating that immune regulators can exert different impacts on the expression of telomerase activity in lymphocytes. Interferon is an important immunomodulator for upregulating host immunity and thus becomes one of the important antiviral drugs approved for the treatment of CHB across the world [26]. In this study, we found that the mean NhTERT mRNA level was significantly lower in each CHB patient group than in healthy control group, which was in agreement with the result studied by Satra et al. [12]. The mean level of NhTERT mRNA in IFN*α*-treated group was remarkably higher than that in CHB control group, and more interestingly, a higher mean level of NhTERT mRNA was shown in the patients with longer duration of IFN*α* treatment. According to the stratified analysis of biochemical, virological, and immunological responses in IFN*α*-treated group, we found that the mean level of NhTERT mRNA in HBeAg loss subgroup was significantly higher compared with the subgroup without, and furthermore, the level in HBeAg loss subgroup was comparable to that in healthy control group; an increased level of NhTERT mRNA could be seen in HBV DNA undetectable subgroup but it did not reach to the statistical significance as compared with HBV DNA detectable subgroup; the mean levels were not of significant difference between ALT normal and abnormal subgroups. The results demonstrated that a lower telomerase activity was expressed in PBMCs of CHB patients, and IFN*α* treatment could obviously upregulate the expression of hTERT mRNA. In view of the multiple cell components existed in PBMCs, therefore we will further investigate whether the change of hTERT expression is derived from the B cells or from the T cells or both, and furthermore, it is also very important to know whether the change is involved in the naïve cells or memory cells, and so forth.

In addition, we can find that there exists a close relationship between the expression of hTERT mRNA in PBMCs and IFN*α* treatment in CHB patients, but the exact mechanism of IFN*α* treatment leading to an increased hTERT mRNA expression is not yet clear. In some researches with tumor cell lines, interferon could both inhibit the expression of hTERT mRNA in leukemia cells or melanoma cells and increase its expression in certain tumor cell lines [27–29]. Recently, Hrdlickova et al. [30] conducted a research on the immune cells, and they found that interferon regulatory factor 4 and 8 correlated with the activation of telomerase promoter in macrophage cell lines, which could increase the transcription of macrophage telomerase and upregulate its activity. In our study, it is not clear whether there is a similar mechanism involved in the increased expression of hTERT mRNA in PBMCs during IFN*α* therapy. However, no matter what the mechanism for the increase of hTERT expression in PBMCs is, we think that the mechanism to upregulate the immune function in CHB patients treated with IFN*α* should at least include the role played by the increased activity of lymphocytic telomerase besides the effects of immune cells and cytokines known previously [31], because lymphocytes with higher telomerase activity attributed to IFN*α* treatment can resist the telomeric erosion, and thus lymphocytes will get a longer life span and more durable ability for cell division, which is the basic precondition for CHB patients to acquire a normally immune response to HBV infection. 

In conclusion, we found that the telomerase activity in PBMCs was closely correlated with the total lymphocyte number and the immune function in CHB patients. The expression of telomerase activity in PBMCs of patients with chronic HBV infection was significantly lower than that in healthy individuals, and IFN*α* treatment could, at least in part, elevate the reduced telomerase activity in PBMCs, particularly in those who achieved HBeAg loss. Therefore, the increased telomerase activity in PBMCs of CHB patients due to IFN*α* therapy could extend the lymphocyte life span, maintain the durable cell division, and promote the immune function, which may be involved in a novel mechanism for IFN*α* antiviral therapy. 

## Figures and Tables

**Table 1:** Clinical baseline characteristics of the study groups.

	IFN*α*-treated Group (*n* = 37)	CHB control group (*n* = 36)	Healthy control group (*n* = 10)	*P*-value
Male	25 (67.6)	28 (77.8)	6 (60)	.56
Median age in year (range)	27.3 (20–48)	28.6 (23–55)	30.6 (24–45)	.68
No. of HBeAg + patients	31 (83.8)	25 (69.4)	N/A	.65
Median ALT (range), IU/L	148.3 (83–288)	132.8 (83–352)	N/A	.61
Median HBV DNA load (range), log copies/ml	7.6 (5.2–9.4)	7.2 (4.8–8.7)	N/A	.72
HBV genotype (no. of patients)	Type B (11)	Type B (8)		.47
	Type C (23)	Type C (25)	N/A	.51
	Other (3)	Other (3)		.97
Median CD3 count (range), 10^6^ cell/L	1138 (971–1493)	1278 (856–1387)	N/A	.72
Median ratio of CD4/CD8 (range)	1.72 (1.13–2.57)	1.58 (1.26–2.63)	N/A	.51

Note. Date are no. (%) of patients, unless otherwise indicated; HBeAg+: positive for hepatitis B e antigen; ALT: alanine aminotransferase; HBV: hepatitis B virus; N/A, not applicable.

**Table 2:** NhTERT mRNA levels in PBMCs of all investigated subjects.

No.	IFN*α* treated group (*n* = 37) NhTERT mRNA	CHB control group (*n* = 36) NhTERT mRNA	Healthy control group (*n* = 10) NhTERT mRNA
1	0.911 × 10^−3^	0.775 × 10^−3^	1.094 × 10^−3^
2	0.985 × 10^−3^	0.930 × 10^−3^	1.204 × 10^−3^
3	1.016 × 10^−3^	0.901 × 10^−3^	1.017 × 10^−3^
4	0.985 × 10^−3^	0.673 × 10^−3^	1.260 × 10^−3^
5	1.096 × 10^−3^	0.792 × 10^−3^	0.959 × 10^−3^
6	1.103 × 10^−3^	0.885 × 10^−3^	1.017 × 10^−3^
7	0.990 × 10^−3^	0.693 × 10^−3^	0.902 × 10^−3^
8	0.989 × 10^−3^	0.989 × 10^−3^	1.069 × 10^−3^
9	1.002 × 10^−3^	0.583 × 10^−3^	0.992 × 10^−3^
10	1.000 × 10^−3^	0.841 × 10^−3^	0.906 × 10^−3^
11	0.983 × 10^−3^	0.749 × 10^−3^	
12	1.058 × 10^−3^	0.927 × 10^−3^	
13	0.914 × 10^−3^	0.980 × 10^−3^	
14	0.994 × 10^−3^	0.736 × 10^−3^	
15	0.989 × 10^−3^	1.010 × 10^−3^	
16	1.034 × 10^−3^	0.875 × 10^−3^	
17	1.042 × 10^−3^	0.757 × 10^−3^	
18	0.991 × 10^−3^	0.954 × 10^−3^	
19	1.061 × 10^−3^	0.907 × 10^−3^	
20	1.220 × 10^−3^	1.001 × 10^−3^	
21	0.924 × 10^−3^	0.880 × 10^−3^	
22	0.952 × 10^−3^	0.887 × 10^−3^	
23	0.994 × 10^−3^	1.088 × 10^−3^	
24	0.800 × 10^−3^	0.790 × 10^−3^	
25	0.980 × 10^−3^	0.708 × 10^−3^	
26	1.018 × 10^−3^	0.941 × 10^−3^	
27	0.809 × 10^−3^	0.917 × 10^−3^	
28	0.967 × 10^−3^	0.655 × 10^−3^	
29	1.088 × 10^−3^	0.593 × 10^−3^	
30	0.791 × 10^−3^	0.916 × 10^−3^	
31	0.932 × 10^−3^	0.942 × 10^−3^	
32	0.717 × 10^−3^	1.088 × 10^−3^	
33	0.751 × 10^−3^	0.702 × 10^−3^	
34	0.863 × 10^−3^	0.968 × 10^−3^	
35	0.943 × 10^−3^	0.965 × 10^−3^	
36	0.770 × 10^−3^	0.859 × 10^−3^	
37	0.809 × 10^−3^		

**Table 3:** Comparison of mean NhTERT mRNA levels between IFN*α*-treated subgroups.

	ALT(IU/L)		HBV DNA (log copies/mL)		HBeAg status*	

	<40	≥40	<3 log	≥3 log	e-Ag (+)	e-Ag (−)
No.	23	14	18	19	17	14
Mean NhTERT	0.967	1.007	1.011	0.950	0.910	1.010
mRNA level (×10^−3^)	±0.105	±0.095	±0.084	±0.105	±0.111	±0.100
*t*-value	1.172		1.955		2.595	
*P*-value	.249		.059		.015	

Note. *subgroup: 31 cases of HBeAg positive CHB; e-Ag (+): without HBeAg loss; (−): with HBeAg loss.
